# The Role of Knowledge and Risk Beliefs in Adolescent E-Cigarette Use: A Pilot Study

**DOI:** 10.3390/ijerph15040830

**Published:** 2018-04-23

**Authors:** Jacob A. Rohde, Seth M. Noar, Casey Horvitz, Allison J. Lazard, Jennifer Cornacchione Ross, Erin L. Sutfin

**Affiliations:** 1 School of Media and Journalism, University of North Carolina at Chapel Hill, Carroll Hall, CB 3365, Chapel Hill, NC 27599, USA; jarohde@unc.edu (J.A.R.); lazard@unc.edu (A.J.L.); 2 Lineberger Comprehensive Cancer Center, University of North Carolina at Chapel Hill, Chapel Hill, NC 27599, USA; horvitz@live.unc.edu; 3 Department of Social Sciences & Health Policy, Division of Public Health Sciences, Wake Forest School of Medicine, Winston-Salem, NC 27157, USA; jcornacc@wakehealth.edu (J.C.R.); esutfin@wakehealth.edu (E.L.S.)

**Keywords:** e-cigarette, vaping, knowledge, beliefs

## Abstract

The use of e-cigarettes and other vaping devices among adolescents is an urgent public health problem due to the concern about adolescent exposure to nicotine. This study examined: (1) adolescents’ knowledge and beliefs about e-cigarette risks; and (2) whether knowledge and risk beliefs were associated with e-cigarette use. *N* = 69 adolescents completed a cross-sectional survey about e-cigarette knowledge, attitudes (i.e., risk beliefs), and behavior (KAB). Nearly half (47%) of the sample reported ever using e-cigarettes. The majority of adolescents knew about many of the risks of e-cigarettes, with no differences between never- and ever-users. However, risk beliefs, such as worrying about health risks of using e-cigarettes, varied across groups. Compared to never-users, e-cigarette ever-users were significantly less likely to worry about e-cigarette health risks, less likely to think that e-cigarettes would cause them negative health consequences, and less likely to believe that e-cigarette use would lead to addiction. In a multivariable logistic regression, prior combustible cigarette use, mother’s education, and addiction risk beliefs about e-cigarettes emerged as significant predictors of adolescents’ e-cigarette use. This study reveals that while knowledge is not associated with adolescent e-cigarette use, risk beliefs do predict use.

## 1. Introduction

The U.S. Surgeon General has identified the increased use of electronic cigarettes (e-cigarettes) and other vaping devices among adolescents as an urgent public health problem [1]. The percentage of high school students who have ever used e-cigarettes or other vaping devices increased from 4.7% in 2011 to 37.7% in 2015, while past 30-day use increased from 1.5% to 16% [1]. Starting in 2014, e-cigarettes became the most commonly used tobacco product by youth and, although use declined from 16% in 2015 to 11.3% in 2016, e-cigarettes remain the most used tobacco product among adolescents [2]. 

A significant concern of adolescent e-cigarette use is addiction. E-cigarette liquid typically contains nicotine, an addictive chemical [3], and exposure to nicotine during adolescence can prime addiction to various other harmful drugs, such as cocaine and methamphetamines [1]. Furthermore, adolescent use of e-cigarettes may give rise to smoking other combustible tobacco products, such as cigarettes [4]. While cigarette smoking among youth has been declining [2], increased use of e-cigarettes has the potential to slow or even reverse these declines [5]. A recent meta-analysis demonstrated that adolescents who use e-cigarettes are at least three times more likely to initiate combustible cigarette use compared to those who do not use e-cigarettes [6]. 

Other health effects of e-cigarettes are also of concern. Chemicals found in e-cigarette aerosol can deposit particles such as nickel, tin, and chromium into the lungs, causing local respiratory toxicity [3]. Dermal exposure to e-cigarette liquid can also cause symptoms such as dizziness, nausea, vomiting, or diarrhea [7]. The long-term health effects of e-cigarette use are also largely unknown. However, several studies suggest e-cigarette use may cause respiratory harm [3,8], increased blood pressure [1], and can interfere with adolescent brain development [1,9]. 

Prevention efforts should be grounded in a careful understanding of adolescents’ knowledge and beliefs about e-cigarettes. Previous investigations have demonstrated that a large majority of adolescents in the U.S. are aware of e-cigarettes [10,11,12,13]; however, few know about their potentially harmful effects [14]. In fact, several studies suggest adolescents tend to believe using e-cigarettes is relatively safe or that they pose minimal health consequences to the body [10,12]. Studies have also shown that few adolescents perceive e-cigarettes to be harmful or toxic [13], and one study demonstrated that some misperceive that e-cigarette use may result in positive health outcomes—e.g., e-cigarette water vapor benefitting lung development [14]. 

Adolescent beliefs about the relative risk of using e-cigarettes have also been examined. Studies show that a majority of adolescents believe using e-cigarettes is far less harmful compared to smoking combustible cigarettes or using other tobacco products [10,15,16]. Furthermore, a recent systematic review suggests that many adolescents are unaware that e-cigarette liquid often contains nicotine [12], also found in combustible cigarettes and other tobacco products. This finding may help explain why young adults tend to believe e-cigarettes are less addictive than combustible cigarettes [17].

While several studies have examined risk beliefs about e-cigarettes, few investigations have examined the role of both e-cigarette knowledge and risk beliefs on e-cigarette use in a single study. The current e-cigarette knowledge, attitude/belief, and behavior (KAB) study was designed to resolve this gap in the literature and contribute to prevention efforts in this area. In the current study, we sought to examine: (1) adolescents’ knowledge and beliefs about e-cigarette risks; and (2) whether knowledge and particular risk beliefs were associated with e-cigarette use. 

## 2. Materials and Methods

### 2.1. Participants

Participants were drawn from a registry of adolescents originally developed from a national phone survey conducted by the Center for Regulatory Research on Tobacco Communication in 2014–2015 [18]. In order to draw an at-risk sample for this pilot study, we mailed letters that included a $2 bill to a sub-sample of adolescents (*n* = 200) who were susceptible to or had used any tobacco product at the time of the 2014–2015 survey. Those interested in participating went to our website and were screened for participation after entering their unique participant code provided in the letter. Inclusion criteria for this pilot study were: currently aged 14–18, had a smartphone, and agreed to send and receive text messages with us. The criteria included having a smartphone because this pilot involved a text-messaging feasibility component that is reported in a separate paper [19]. Two weeks after the original letter was sent, we mailed a second letter (with no cash) to adolescents who had not yet taken the screening survey. These recruitment efforts yielded 88 prospective participants who took the screening survey. Five were ineligible (one did not have a smartphone, four did not want to send and receive multiple text messages) and 14 were eligible but chose not to enroll. Thus, our final sample was *N* = 69. 

### 2.2. Measures

*E-cigarette knowledge*. E-cigarette knowledge was assessed using six items. Participants were asked if they knew that e-cigarettes: (1) usually contain nicotine, an addictive chemical; (2) use liquid that contains harmful chemicals; (3) may harm teen brain development; (4) have unknown long-term health effects; (5) are not risk-free; and (6) use liquid that is made from tobacco. Participants answered “true,” “false,” or “don’t know.” Responses were dichotomized into correct (True—1) versus other (False or don’t know—0) answers and summed to create a composite knowledge score, with a higher score indicating greater knowledge.

*Tobacco product susceptibility.* E-cigarette susceptibility was assessed using five items [20,21]; participants were asked, “do you think that…” followed by example items such as “you will use an e-cigarette or other vaping device soon?” and “if one of your best friends were to offer you an e-cigarette or other vaping device, would you use it?” Cigarette susceptibility was assessed using the latter ‘best friend’ item only. Responses were on a four-point scale from “definitely no” to “definitely yes.” Participants were susceptible if they answered anything other than “definitely no” to any of the questions.

*Risk beliefs—perceived risks.* Perceived risks was adapted from an existing scale [22] and began with the stem, “If I were to use an e-cigarette or other vaping device, I would…” and initially contained 10 items. An exploratory factor analysis of the items revealed that they factored into three dimensions: health worry (three items—e.g., “worry about my health”), health consequences (three items—e.g., “harm my lungs”), and addiction (two items—e.g., “get addicted”). Two items loaded complexly (i.e., loaded on several different factors) and were dropped. Responses were on a five-point scale from “definitely wouldn’t” to “definitely would.” Coefficient alpha of the full 8-item scale was α = 0.86, while for the subscales was as follows: health worry (α = 0.89), health consequences (α = 0.87), and addiction (*r* = 0.66). 

*Risk belief—perceived relative risk.* Perceived relative risk was measured using one item that asked participants to rate the risks of using e-cigarettes compared to smoking combustible cigarettes on a five-point scale ranging from “much less harmful” to “much more harmful”.

*Tobacco product use.* E-cigarette use was assessed by asking participants if they had ever used e-cigarettes or other vaping devices in their lifetime (ever use) and in the past 30 days (current user). Cigarette smoking [23] was assessed by asking if participants had ever tried smoking cigarettes (ever use) and if they now smoke some days or every day (current smoker). Other tobacco product use was assessed by having participants select other tobacco products they had ever used in their lifetime from a list. Other tobacco products included: chewing tobacco; cigarillos; filtered cigars or little cigars; waterpipes; moist snuff; traditional cigars; snus; and pipe filled with tobacco.

### 2.3. Procedure

Adolescents who were eligible and chose to enroll in the study were automatically directed to an assent form online. Parental informed consent was waived since participants’ parents had already given consent for the earlier national phone survey study. After assent, participants conducted a mobile phone verification task. Once verified, the website automatically redirected participants to the e-cigarette survey. Upon completion of the survey, participants were texted a $10 Amazon gift code. The University of North Carolina at Chapel Hill IRB approved all procedures used in this study (IRB study #17-0644).

### 2.4. Data Analysis

Descriptive statistics were used to characterize e-cigarette knowledge and beliefs. Chi-square tests were used to compare e-cigarette never- and ever-users for variables with dichotomous outcomes, and independent samples *t*-tests were used for continuous outcomes. A multivariable logistic regression analysis was used to predict ever use of e-cigarettes (0 = never used, 1 = ever used). We first ran bivariate analyses using a probability threshold of *p* < 0.10 on all primary measures and covariates to determine which variables to include in the regression analysis. All variables associated with e-cigarette ever use at *p* < 0.10 were included in the logistic regression analysis. All analyses were computed using SPSS version 23 (SPSS Inc., Chicago, IL, USA) and RStudio (R Foundation for Statistical Computing, Vienna, Austria). All tests met necessary statistical assumptions.

## 3. Results

### 3.1. Participant Characteristics

Of the 69 baseline survey participants, 48% were male, 48% female, and 4% were gender non-conforming. The mean age was 16.33 (*SD* = 0.89). The majority of participants were White (81%), followed by African American (11%). Seven percent were Hispanic. The majority of participants were in 11th (29%) or 12th (41%) grade. 

For descriptive purposes, we classified participants into four mutually exclusive categories of e-cigarette use and susceptibility: 17% percent were current e-cigarette users (past 30 days), 30% had ever used e-cigarettes but were not current users, 41% were susceptible never-users of e-cigarettes, and 12% were non-susceptible never-users. Thirty-one percent of the sample had ever smoked combustible cigarettes and between 6% and 23% had ever used other tobacco products such as chewing tobacco and little cigars.

Nearly half (47%) of the sample reported having ever used e-cigarettes in their lifetime. Of those, the majority were male (61%) and nearly half (48%) reported ever or currently smoking combustible cigarettes. Among the never e-cigarette users, females (56%) made up the majority, and only 14% of these e-cigarette never-users had ever smoked combustible cigarettes (Table 1).

### 3.2. Knowledge and Beliefs about E-Cigarettes

Knowledge about e-cigarette risks among the full sample varied by topic: 83% knew that e-cigarettes usually contain addictive nicotine, 74% knew that e-cigarettes have unknown long-term health effects, 67% knew that e-cigarette liquid contains harmful chemicals, and 59% knew that e-cigarettes are *not* risk free. Only 49% knew that e-cigarettes may harm teen brain development and 45% of participants knew that e-cigarette liquid is made from tobacco. No significant differences were found between never- and ever-users of e-cigarettes on a composite knowledge score or any of the individual knowledge items (see Table 2).

Compared to never-users, e-cigarette ever-users were significantly less likely to worry about the health risks of e-cigarettes (*M* = 3.18 vs. *M* = 3.80, *p* = 0.031), less likely to think that e-cigarettes would cause them negative health consequences (*M* = 3.06 vs. *M* = 3.56, *p* = 0.049), and less likely to believe that e-cigarette use would lead to addiction (*M* = 2.12 vs. *M* = 3.10, *p* < 0.001; see Table 2). Although never-users rated e-cigarettes higher on the perceived relative risk scale compared to ever-users, this difference was not statistically significant. Both groups viewed e-cigarettes as less harmful than combustible cigarettes (a 3 on the scale indicated equally harmful). 

### 3.3. Predicting E-Cigarette Use

We computed a multivariable logistic regression model with covariate and predictor variables. Controlling for the covariates of gender, cigarette ever use, and mother’s education, we examined whether e-cigarette perceived risks (worry, consequences, and addiction) and perceived relative risk predicted e-cigarette use. Results indicated that only addiction risk beliefs were associated with e-cigarette use—i.e., participants who believed that e-cigarette use leads to addiction were less likely to have ever used e-cigarettes (*p* < 0.05). Also, the cigarette ever use covariate was significant and had a large effect; participants who had ever smoked combustible cigarettes were over four-and-a-half times more likely to be ever-users of e-cigarettes (*p* < 0.05). Finally, the covariate mother’s education was significant, indicating that participants with mothers who had at least a bachelor’s degree were less likely to have ever used e-cigarettes (*p* < 0.05). No other variables were significantly associated with e-cigarette use (see Table 3). 

## 4. Discussion

The purpose of this KAB study was to examine how adolescents’ knowledge and beliefs about e-cigarettes were associated with e-cigarette use to inform prevention efforts in this area. We found that the majority of adolescents knew about many of the risks of e-cigarettes, but that knowledge played little or no role in their e-cigarette use behavior. This finding is consistent with the proposition that knowledge may be a necessary but not sufficient condition for behavior change. Indeed, knowledge is conspicuously absent from most theories of health behavior [24], reflecting the fact that beliefs often play a much larger role in behavior and behavior change than knowledge [25]. 

We also found that adolescents who had ever used e-cigarettes were more likely than never-users to discount the risks of addiction that e-cigarettes pose. This finding reveals a paradox in that e-cigarette users were knowledgeable that e-cigarettes usually contain addictive nicotine, but did not believe they will become addicted. This is troubling because many studies have shown that adolescents are especially vulnerable to the effects of nicotine [1,26], and addiction to nicotine may increase use of other tobacco products such as combustible cigarettes [4,6]. Furthermore, studies show that more than three out of five people who try smoking become daily smokers [27], and of those lifelong smokers, roughly 90% begin smoking when they are under the age of 18 [28]. This suggests that adolescents do not entirely understand or appreciate the risk of addiction [29], strongly suggesting the need to increase health literacy among adolescents regarding the nature and serious consequences of addiction. 

The Food and Drug Administration (FDA) recently announced that, beginning in 2018, all e-cigarette advertisements and packaging are required to affix a warning label stating the following: “WARNING: This product contains nicotine. Nicotine is an addictive chemical” [30]. While this represents an important step in educating the public about e-cigarettes, our results suggest that adolescents are largely aware of this information—i.e., more than 80% of our sample reported knowing that e-cigarettes usually contain addictive nicotine. In the future, the FDA may wish to expand labeling requirements to include more information about the potential negative consequences of nicotine exposure and addiction.

Finally, our study showed that adolescents generally view e-cigarettes as less harmful than combustible cigarettes, supporting several previous investigations [10,15,16]. However, recent studies have shown that adolescents cite the lower relative harm of vaping compared to smoking combustible cigarettes as one of the reasons why they use e-cigarettes [1,31,32]. Therefore, future messaging and prevention efforts must strike a careful balance in communicating that while e-cigarettes are likely to be less harmful than combustible cigarettes, they are *not* risk free and may pose particular harm for adolescents. 

Our study had some limitations. Although we drew our sample from a nationally representative sample, our pilot study sample was not nationally representative and we had a modest sample size, potentially reducing statistical power. The fact that we found several significant effects, however, may attest to the robustness of the observed effects. Future studies should confirm and extend the work conducted here in larger samples. In addition, our study was cross-sectional and therefore we cannot make causal conclusions. Further work should use longitudinal designs to examine the influence of knowledge and beliefs on e-cigarette use over time, as well as how knowledge and beliefs change in response to the changing communication landscape around e-cigarettes and other vaping devices.

## 5. Conclusions

In conclusion, our KAB study shows that adolescents are generally knowledgeable about e-cigarette risks but that this knowledge is not associated with e-cigarette use behavior. Rather, addiction risk beliefs about e-cigarette use were strongly associated with e-cigarette use. Health communication messages and campaigns should thus attempt to increase perceptions of the likelihood and severity of addiction through product warnings and public media campaigns, including through the Food and Drug Administration’s national youth prevention *The Real Cost* campaign [33]. More generally, our findings suggest the need for greater health literacy about the harms of e-cigarettes for adolescents, especially addiction. Future studies might also explore addiction risk beliefs in more depth, examining perceptions of the relative addictiveness and consequences of addiction to combustible cigarettes and e-cigarettes.

## Figures and Tables

**Table 1:** Participant characteristics, *N* = 69 adolescents.

	E-Cigarette Ever-Users *n* (%) or *M* ± *SD*	E-Cigarette Never-Users *n* (%) or *M* ± *SD*	Total *n* (%) or *M* ± *SD*
Gender			
Male	20 (61%)	13 (36%)	33 (48%)
Female	13 (39%)	20 (56%)	33 (48%)
Non-conforming	0 (0%)	3 (8%)	3 (4%)
Age, years	16.36 ± 0.90	16.31 ± 0.89	16.33 ± 0.89
Year in school			
9th grade	4 (12%)	3 (8%)	7 (10%)
10th grade	5 (15%)	5 (14%)	10 (14%)
11th grade	9 (27%)	11 (31%)	20 (29%)
12th grade	13 (39%)	15 (42%)	28 (41%)
Not currently in school	2 (6%)	2 (6%)	4 (6%)
Race ^1^			
White	30 (91%)	31 (86%)	61 (81%)
Black or African American	4 (12%)	4 (11%)	8 (11%)
Asian	3 (9%)	1 (3%)	4 (5%)
Other	1 (3%)	1 (3%)	2 (3%)
Hispanic	4 (12%)	1 (3%)	5 (7%)
Mother’s education			
High school or less	8 (24%)	3 (8%)	11 (16%)
Some college or associate’s	7 (21%)	3 (8%)	10 (14%)
Bachelor’s degree	8 (24%)	19 (53%)	27 (39%)
Graduate degree	9 (27%)	11 (31%)	20 (29%)
Not reported	1 (3%)	0 (0%)	1 (2%)
Father’s education			
High school or less	18 (55%)	10 (28%)	28 (41%)
Some college or associate’s	4 (12%)	8 (22%)	12 (17%)
Bachelor’s degree	5 (15%)	8 (22%)	13 (19%)
Graduate degree	5 (15%)	9 (25%)	14 (20%)
Not reported	1 (3%)	1 (3%)	2 (3%)
E-cigarette use			
E-cigarette users (past 30 days)	12 (36%)	--	12 (17%)
E-cigarette users (ever, not past 30 days)	21 (64%)	--	21 (30%)
Susceptible never-user ^2^	--	28 (78%)	28 (41%)
Non-susceptible never-user ^2^	--	8 (22%)	8 (12%)
Cigarette smoking			
Cigarette smoker (current)	2 (6%)	0 (0%)	2 (3%)
Cigarette smoker (ever, not past 30 days)	14 (42%)	5 (14%)	19 (28%)
Susceptible never-user ^3^	2 (6%)	7 (19%)	9 (13%)
Non-susceptible never-user ^3^	15 (46%)	24 (67%)	39 (56%)
Tobacco product use (lifetime)			
Chewing tobacco	11 (33%)	5 (14%)	16 (23%)
Cigarillos, Filtered cigars, or little cigars	13 (39%)	1 (3%)	14 (20%)
Waterpipe	10 (30%)	2 (6%)	12 (17%)
Moist Snuff	8 (24%)	3 (8%)	11 (16%)
Traditional cigars	6 (18%)	2 (6%)	8 (12%)
Snus	4 (12%)	0 (0%)	4 (6%)
Pipe filled with tobacco	4 (12%)	0 (0%)	4 (6%)

Note: ^1^ Participants were allowed to select more than one race; ^2^ When e-cigarette susceptibility is computed using only the “best friend” item, the results are *n* = 22 (61%) susceptible never-user and *n* = 14 (39%) non-susceptible never-user; ^3^ Assessed using single “best friend” item only.

**Table 2:** Knowledge and beliefs about e-cigarettes, *N* = 69 adolescents.

	Ever-Users *M* ± *SD* or *n* (%)	Never-Users *M* ± *SD* or *n* (%)	*p*
Knowledge	3.52 ± 1.46	4.00 ± 1.84	0.23
Usually contains nicotine, an addictive chemical	28 (85%)	29 (81%)	0.76
Liquid contains harmful chemicals	20 (61%)	26 (72%)	0.32
May harm teen brain development	15 (45%)	19 (53%)	0.63
Have unknown long-term health effects	25 (76%)	26 (72%)	0.79
Are *not* risk-free	16 (48%)	25 (69%)	0.09
Use liquid that is made from tobacco	12 (36%)	19 (53%)	0.23
Risk beliefs—Perceived risks	2.79 ± 0.87	3.48 ± 0.83	**0.001**
Health worry	3.18 ± 1.20	3.80 ± 1.11	**0.031**
Health consequences	3.06 ± 0.94	3.56 ± 1.10	**0.049**
Addiction	2.12 ± 1.25	3.10 ± 0.98	**<0.001**
Risk belief—Perceived relative risk	1.94 ± 0.83	2.31 ± 0.82	0.07

Note: Statistically significant *p* values in bold.

**Table 3:** Logistic regression predicting ever use of e-cigarettes, *N* = 68 adolescents.

	Adjusted Odds Ratio	95% Confidence Interval
Gender		
Females/non-conforming	REF	
Male	2.35	(0.60–9.23)
Cigarette ever use		
Never used	REF	
Has used	4.90 *	(1.23–19.53)
Mother’s education		
Has less than a bachelor’s	REF	
Has at least a bachelor’s degree	0.24 *	(0.06–1.00)
Risk beliefs—Perceived risks		
Health worry	1.05	(0.57–1.96)
Health consequences	1.21	(0.58–2.55)
Addiction	0.46 *	(0.25–0.83)
Risk belief—Perceived relative risk	1.06	(0.44–2.56)

Note: REF = reference group; * *p* < 0.05; one participant excluded from analysis due to missing data.
